# Auricular Acupressure for Managing Postoperative Pain and Knee Motion in Patients with Total Knee Replacement: A Randomized Sham Control Study

**DOI:** 10.1155/2012/528452

**Published:** 2012-07-10

**Authors:** Ling-hua Chang, Chung-Hua Hsu, Gwo-Ping Jong, Shungtai Ho, Shiow-luan Tsay, Kuan-Chia Lin

**Affiliations:** ^1^Department of Nursing, Landseed Hospital, Pingjhen City 32449, Taiwan; ^2^Department of Nursing, Hsin Sheng College of Medical Care and Management, Pingjhen City 32462, Taiwan; ^3^Institute of Traditional Medicine, National Yang-Ming University, Taipei 11221, Taiwan; ^4^Linsen (Chinese Medicine) Branch, Taipei City Hospital, Taipei 10844, Taiwan; ^5^Division of Internal Cardiology, Taichung Armed Forces General Hospital, Taichung 41152, Taiwan; ^6^Basic Medical Science, Centeral Taiwan University of Science and Technology, Taichung 40601, Taiwan; ^7^Department of Anesthesiology, National Defense Medical Center, Taipei 11490, Taiwan; ^8^Department of Anesthesiology, Taipei Veterans General Hospital, Taipei 11217, Taiwan; ^9^College of Nursing, National Taipei University of Nursing and Health Sciences, Taipei 11214, Taiwan

## Abstract

*Background*. Postoperative pain management remains a significant challenge for all healthcare providers. A randomized controlled trial was conducted to examine the adjuvant effects of auricular acupressure on relieving postoperative pain and improving the passive range of motion in patients with total knee replacement (TKR). *Method*. Sixty-two patients who had undergone a TKR were randomly assigned to the acupressure group and the sham control group. The intervention was delivered three times a day for 3 days. A visual analog scale (VAS) and the Short-Form McGill Pain Questionnaire were used to assess pain intensity. Pain medication consumption was recorded, and the knee motion was measured using a goniometer. *Results*. The patients experienced a moderately severe level of pain postoperatively (VAS 58.66 ± 20.35) while being on the routine PCA. No differences were found in pain scores between the groups at all points. However, analgesic drug usage in the acupressure group patients was significantly lower than in the sham control group (*P* < 0.05), controlling for BMI, age, and pain score. On the 3rd day after surgery, the passive knee motion in the acupressure group patients was significantly better than in the sham control group patients (*P* < 0.05), controlling for BMI. *Conclusion*. The application of auricular acupressure at specific therapeutic points significantly reduces the opioid analgesia requirement and improves the knee motion in patients with TKR.

## 1. Introduction

Knee osteoarthritis frequently occurs in elderly people. The overall prevalence of knee osteoarthritis is 23.9%, occurring in 21.0% of men and 27.3% of women [1]. Knee-replacement surgery is frequently performed and is highly successful with respect to pain relief and the improvement in knee function in people with advanced knee osteoarthritis [2]. Postoperatively, patients undergoing total knee replacement would experience significant pain, which can limit their progression in the range of knee motion and ambulatory status.

Despite the development of new pain control methods and medication and the adoption of guidelines for pain care by many hospitals, postoperative pain is still a problem for many patients. A substantial proportion of total knee replacement (TKR) patients experience severe postoperative pain during the early recovery period [3]. Improper pain control not only increases the burden on many organs but also limits the patient's activity, increases the postoperative morbidity, affects the physical recovery and the emotional state of the patient after surgery, and is more likely to extend the length of the stay and increase the medical costs [4–8].

Generally, the clinical management of postoperative pain following TKR mainly involves the use of opioids through patient-controlled analgesia (PCA) to provide the patient with satisfactory pain relief [9]. Given the inherent side effects and limitations of conventional opioids, studies have been conducted to investigate the effective use of non-opioids or even nonpharmacological treatment modalities. Acupuncture is a nonpharmacological treatment modality for the management of postoperative pain. Recent studies have found that the application of acupuncture to auricular acupoints is effective in producing analgesia [10–13].

Using auricular acupuncture to reduce postoperative pain is promising but not compelling based on a systematic review [14]. Auricular acupuncture using a combination of auricular Shenmen and other relevant points can relieve postoperative pain after orthopedic surgery [15–18]. Shenmen (TF4) is at the upper part of the posterior 1/3 of the fossa and has analgesic, sedative, and anti-inflammatory effects [19]. In clinical setting, Subcortex (AT4) is frequently applied for managing pain and pain-related anxiety [20]. AT4 is at the medial side of antitragus and serves to regulate the cerebral cortex function.

Auricular acupressure is similar to acupuncture, but to our knowledge, there had been no randomized controlled trial (RCT) assessing the effectiveness of auricular acupressure for pain management after TKR surgery. Moreover, routine acupuncture in a clinical setting has its limitations. First, many hospitals are not equipped with an acupuncturist or related resources. Second, the cost for acupuncture is expensive for many patients. Auricular acupressure is a noninvasive intervention and requires less technical expertise. In addition, it may be more acceptable to patients compared with acupuncture. Hence, this study aimed to examine the adjuvant effects of auricular acupressure on reducing analgesic drug requests and improving the range of motion in the knee in postoperative patients after total knee replacement.

## 2. Methods

Ethical approval was obtained from the Hospital Authority of Taiwan and the National University of Nursing and Health Sciences. Sixty-two patients scheduled to undergo an elective total knee replacement were recruited from August 1st, 2010 to March 31, 2011. The inclusion criteria were the following: (i) degenerative arthritis and having total knee replacement surgery, (ii) use of patient-controlled analgesia (PCA) after surgery and an American Society of Anesthesiologist physical status of Classes I–III, (iii) ability to communicate in Taiwanese or Mandarin, and (iv) agreement to participate in this study.

Patients were excluded if they suffered from pain or injuries in the ears, had a history of neurological disorders that could alter their perception of pain, a prior experience with auriculotherapy, malignant tumors, a serious chronic illness, mental illness, or drug/alcohol addiction, or were unable to give consent or follow instructions during the data collection process.

All of the patients received an operation under standardized general anesthesia protocols. The patients received routine pain management at the end of the operation: the intravenous PCA was connected to an IV line and set to deliver a bolus of 1 mg morphine with a lockout interval of 5 minutes and a 4-hour maximum morphine dose of 10 mg.

### 2.1. Experimental Procedures

**Figure 1:**
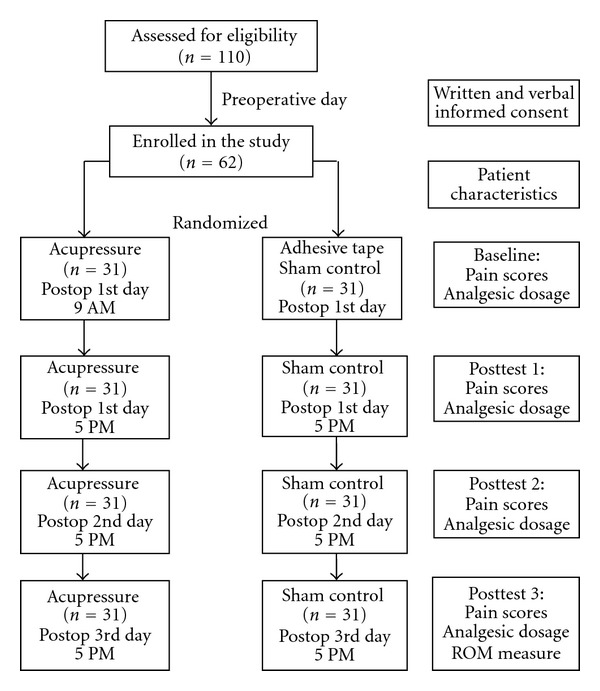
The study procedures and number of patients.

Patients who met the study criteria were interviewed by the investigator before the operation; the purposes of the study were explained, and written consent to participate in the study was then obtained. Throughout the study period, one acupressure therapist performed the auricular acupressure, and the outcome data were collected by a research assistant who had been trained and was blinded to the patient's group. The physicians, anesthesiologists, and nurses had no previous knowledge of acupressure and were blinded to the patients' group allocation. The data collection processes are shown in Figure 1.

**Figure 2:**
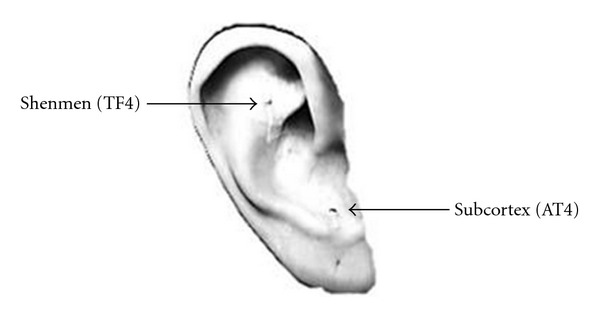
The auricular acupoints chosen for acupressure.

The study, which was conducted at a regional teaching hospital in central Taiwan, used a sham control group and double-blinded experimental design. Sixty-two patients were recruited in our study during an eight-month period. Based on computer-generated codes, block randomization was used with a block size of 4. The patients were randomly assigned into the auricular acupressure or sham control groups. The auricular acupressure therapy was delivered by the therapist, who was an advanced practice nurse and had been accredited for practicing acupressure. The auricular acupressure involved embedding the magnetic beads within skin-colored adhesive tape that was placed on the auricular acupoints and retained in situ for 3 days (Figure 2). The choice of Shenmen (TF4) and subcortex (AT4) acupoints was based on clinical reports [15–18, 21] and the TCM physician's recommendation. Acupressure then was applied by repeatedly pressing the acupoints with the fingertips for 3 minutes per point, 3 times per day (9 AM, 1 PM, 5 PM). The last treatment was given on the third day after surgery at 5 PM. To validate the auricular acupressure, two Chinese medicine practitioners confirmed the acupoints and the acupressure protocol.

The patients in the sham control group received regular care and also received the same skin-colored adhesive tape placed on the acupoints but did not receive any massage or acupressure. The sham control patients were given auricular acupressure after the data collection was completed.

The sample size was estimated based on the data of the pilot study. With an effect size *f* of 0.84, an *α* error probability of 0.05 and data collection performed in triplicate, a sample size of 62 would achieve statistical power up to 0.90.

The pain assessment was conducted using the visual analog scale (VAS) and the Short-Form McGill Pain Questionnaire (SF-MPQ), both of which have good reliability [22]. The VAS was used to measure the pain perceived by the patients through a vertical line scale ranging from 0 to 100 mm. A score of 0 indicates “not painful at all”, and a score of 100 indicates “the most severe pain ever experienced”. Higher scores indicate more severe degrees of perceived pain [23]. The Cronbach's *α* was 0.83 in this study.

The SF-MPQ was modified from the MPQ by Melzack in 1987 [24]. The scale covers the nature of pain and the quantity of pain. The sensory dimension comprises 11 questions, the affective dimension comprises 4 questions, and the present pain intensity (PPIS) dimension comprises one question. The SF-MPQ has been used to assess postoperative pain, labor pain, bone pain, and muscle pain. Similar to the McGill Pain Questionnaire (MPQ), the short form of the questionnaire has good validity, and the correlation between the two reaches 0.65~0.94 [25]. This study adopted the Chinese version of the SF-MPQ, which has been shown to have good internal reliability (Cronbach's *α* range, 0.78 to 0.83) [26]. Cronbach's *α* was 0.76 in this study.

The range of motion is measured with the flexion mobility of the knee at day 3 after the surgery using the goniometer integrated in the dynamometer [27]. Non-weight-bearing passive ROM values were obtained with the patient in the supine position to allow free hip flexion.

### 2.2. Statistical Analysis

Data management and analyses were conducted using the SPSS for Windows statistical package, version 17.0. The patients' demographic profiles and baseline data were compared using the *t*-test, *χ* ^2^, or one-way analysis of variance (ANOVA). A generalized estimation equation (GEE) modeling was applied to investigate the effect of auricular acupressure for the outcomes of pain and opioid consumption. A *t*-test was used to compare differences in knee motion between the acupressure and sham control groups. A *P* value of less than 0.05 was considered to indicate statistical significance.

## 3. Results

A total of 62 patients completed the study, 53 women and 9 men, with a mean age of 70.98 (range 46–88) years. The operations included 31 left knees and 31 right knees and were performed by 2 surgeons. There were no differences between the acupressure and sham control groups in terms of the demographic and clinical characteristic data obtained at the baseline (all *P* > .05) (Table 1). None of the patients who received auricular acupressure showed any complications or adverse reactions after the therapy.

### 3.1. The Changes in the Pain Scores

**Figure 3:**
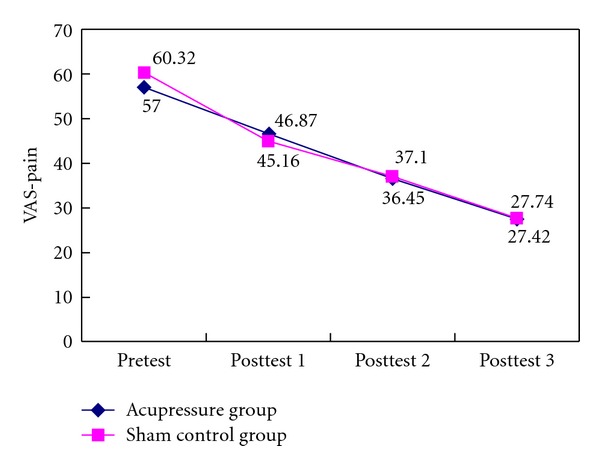
Pain scores (VAS) between the groups over time.

The descriptive data of the postoperative pain scores (VAS-pain and Short-form McGill Pain Questionnaire) reported by the patients are shown in Table 2. Overall, the patients experienced moderately severe pain on the 1st day after surgery (VAS 58.66 ± 20.35) while being on the routine patient-controlled analgesia. However, the pain scores decreased gradually over time (Figure 3) but did not reach significant between-group differences in either of the pain scores (*P* > .05).

### 3.2. The Changes in Pain Medication Use

**Figure 4:**
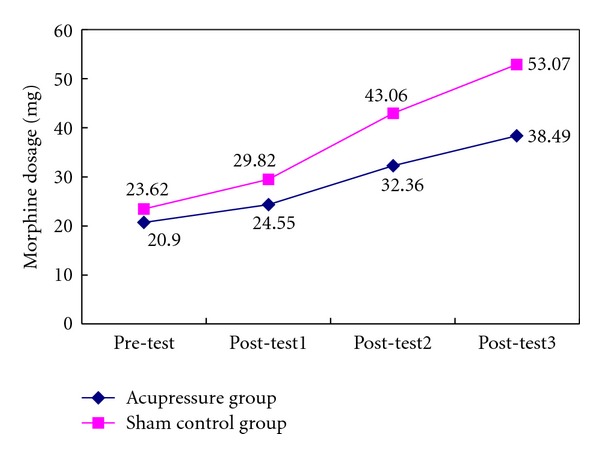
Analgesic morphine usage between the groups over time.

The results of the analgesic (morphine) usage of the patients are summarized in Figure 4. The analgesic drug usage (38.49 ± 15.53 mg of morphine) in the acupressure group patients was significantly lower than in the sham control group patients (53.07 ± 19.90 mg) (*P* < 0.05). Additionally, after controlling for BMI, age, and pain scores (VAS) in the generalized estimated equation model analysis, analgesic drug consumption in the acupressure group patients remains significantly lower than in the sham control group (*P* = .002) patients over time (Table 3). The dose of morphine in the sham control group was 8.38 times higher than in the acupressure group.

### 3.3. The Changes in the Passive Flexion Motion of the Knee

On the 3rd day after surgery, the passive knee flexion motion in the acupressure group patients (71.68 ± 6.90°) was significantly better than in the sham control group patients (66.94 ± 7.15°) (*P* = .01). The results of Pearson's correlation analysis found a negative correlation between the knee flexion motion and BMI (*P* < 0.05). Therefore, further regression analysis was conducted, and after controlling for BMI, the results remained significant between the groups (*P* = .02).

## 4. Discussion

Our patients reported postoperative pain as moderately severe during the 1st day (baseline) following TKR while the patients were on PCA for pain management. This result corresponded with previously reported results [1] and those of Norkin et al. [27], who reported that patients are still in pain while being treated with analgesic drugs and techniques, indicating that the management of postoperative pain remains a challenge for medical practitioners. Subsequently, alongside the conventional opioid analgesics, an adjuvant treatment involving a nonpharmacological modality may offer some benefits in the management of postoperative pain in TKR patients.

Similar to acupuncture, we found that 3 days of auricular acupressure can significantly lower the postoperative opioid consumption after a total knee replacement surgery. Our results showed that the morphine consumption in the sham control group patients was 8.38 times higher than that in the acupressure group. This finding is consistent with that of previous studies, which showed that auricular acupuncture is effective in managing postoperative pain [14, 15]. Usichenko et al. [15–17] demonstrated that auricular acupuncture significantly reduced the consumption of analgesics by patients after they had undergone a total hip arthroplasty and an ambulatory knee arthroscopy.

The analgesic effect of acupressure in the reduction of required postoperative morphine can most likely be explained through the stimulation of acupoints, which can adjust organs, correct imbalances of Qi, stabilize the body, strengthen the functions, and cure diseases [28]. Acupoints stimulation also increases endorphin secretion and serotonin production, thereby suppressing the transmission of pain messages and its perception [28].

Our present findings illustrate that auricular acupressure applied to the Shenmen (TF4) and Subcortex (AT4) acupoints can also improve the range of motion of the knee after the operation. This effect can be explained primarily because auricular acupressure produced an analgesic effect, allowing patients to have a better compliance with the treatments designed to improve the range motion in the knee. A literature search revealed no acupressure studies and only one acupuncture study in a TKR population. Tsang et al. concluded that acupuncture was no better than sham acupuncture in pain relief and in improving the range of knee motion in patients who had undergone bilateral total knee arthroplasty [29]. Nevertheless, that study suffered from small sample size. Thus, more studies need to be conducted and documented to support the effectiveness of acupuncture or acupressure in total knee replacement patients.

The acupressure procedure was safe and easy to perform under the perioperative clinical conditions using finger press and massage on the auricular acupoints after the operation. The study protocol was based on the experts' recommendations for the practice of acupuncture. Although the randomization in the groups was successful as indicated by the balance regarding age, gender, and BMI, which can confound the postoperative analgesic requirement, we improved the control over BMI in the statistical analysis to increase the validity of our results. Our double-blinded study design minimized the potential biases. The blinding of patients and data collectors was adequate, which strengthened the credibility of the main result.

We selected a randomized sham control design and used adhesive tape on the acupoints as the sham control. We also provided acupressure to the sham control group patients at the end of this study. With this design, all participants eventually received the therapeutic auricular acupressure. Our design may avoid the ethical dilemma of withholding a potentially helpful treatment for half of the participants. Noticeably, our study had no actual placebo group for comparing the study effects. According to TCM theory, the body contains 14 meridians (‘‘Jing”, like boulevards) that bear countless branches (“Luo”, like byways). As the theory notes, finding a body site without the influence of any Jing and Luo would be difficult. Considering that most of the meridians are densely distributed, especially in the ear, massage anywhere on the body can produce the same therapeutic effect [28]. Consequently, using a nonacupoints placebo group in this study may be inappropriate. Thus, our design has no true placebo group, and the sham control group was used to try to distinguish the auricular acupressure effects from the psychological effects.

A potential limitation of our research is that those who chose to be involved in the trial were limited to one hospital, and the findings may therefore not be indicative of the changes likely to occur with the general TKR population. Furthermore, the followup period of 3 days after the TKR surgery was short, and we have no way to determine the long-term effects of auricular acupressure. Furthermore, the dose of acupressure may have been inadequate; for example, more frequent treatments or a different treatment protocol may have generated different results. Given that reasonable evidence on the course effectiveness has been established in the current study, further research should focus on longer-term followup and collecting data from multiple centers to determine whether the changes and positive effects of acupressure are maintained over time.

 In conclusion, a program of auricular acupressure applied to therapeutic auricular acupoints significantly reduces the opioid analgesic consumption and improves the passive range motion of the knee postoperatively after total knee replacement. However, both groups of patients experienced a similar degree of pain, based on their reported scores, while on the routine PCA for pain management.

## Figures and Tables

**Table 1:** Demographic and clinical characteristics of the study groups.

	Acupressure (*n* = 31)	Sham control (*n* = 31)	*P* value (between group)
Age^a ^ (range 46–88)	71.23 ± 7.09	70.74 ± 8.09	.803
BMI^a^	27.19 ± 3.08	28.73 ± 4.14	.103
Gender^b^			
Male	3 (9.7)	6 (19.4)	.473
Female	28 (90.3)	25 (80.6)	
Religious belief ^b^			
Yes	27 (87.1)	23 (74.2)	.335
No	4 (12.9)	8 (25.8)	
Marital status^b^			
Married	18 (58.1)	23 (74.2)	.283
Widowed/separated	13 (41.9)	8 (25.8)	
Education^b^			
Illiterate	11 (35.5)	9 (29.0)	.786
Primary school or higher	20 (64.5)	22 (71.0)	
Occupation^b^			
No	29 (93.5)	24 (77.4)	.147
Yes	2 (6.5)	7 (22.6)	
Joint replacement history^a^			
Yes	5 (16.1)	4 (12.9)	1.00
No	26 (83.9)	27 (87.1)	
Surgical site^b^			
Left	14 (45.2)	17 (54.8)	.612
Right	17 (54.8)	14 (45.2)	

^a^Independent *t*-test; ^b^Chi-Square test.

**Table 2:** Changes in pain scores between the groups over time.

	All	Acupressure	Sham control	*P* value
	(*n* = 62)	(*n* = 31)	(*n* = 31)	
	Mean ± SD	Mean ± SD	Mean ± SD	
VAS				
Baseline	58.66 ± 20.35	57.00 ± 17.83	60.32 ± 22.77	.525
Posttest 1	46.02 ± 18.32	46.87 ± 17.05	45.16 ± 19.77	.717
Posttest 2	36.77 ± 16.02	36.45 ± 13.74	37.10 ± 18.25	.876
Posttest 3	27.58 ± 13.96	27.42 ± 12.24	27.74 ± 15.70	.928
SF-MPQ				
Baseline	12.85 ± 5.93	12.26 ± 5.79	13.45 ± 6.11	.433
Posttest	5.18 ± 3.84	5.16 ± 4.08	5.19 ± 3.66	.974
Pain PPI				
Baseline	2.95 ± 1.02	2.74 ± .89	3.16 ± 1.10	.104
Posttest	1.34 ± .63	1.23 ± .50	1.45 ± .72	.157

VAS: visual analog scale for pain; SF-MPQ: Short-Form McGill Pain Questionnaire; Pain PPI: present pain intensity score of SF-MPQ.

**Table 3:** Generalized estimated equation model of the effects of auricular acupressure on pain medication use.

	*β*	Standard errors	Wald chi-squared	*P* value
Intercept	54.16	18.36	8.70	.003
Group (sham control versus acupressure)	8.38	2.73	9.42	.002
Baseline versus Posttest 1	−23.32	2.00	135.47	<.001
Posttest 1 versus Posttest 2	−18.42	1.64	126.25	<.001
Posttest 2 versus Posttest 3	−7.98	1.43	31.27	<.001
BMI	.05	.38	.02	.90
Age	−.19	.18	1.12	.29
Pain score (VAS)	.02	.05	.10	.75

## References

[1] Pereiraya D, Peleteiroyza B, Araújoyza J, Brancoxa J, Santoska RA, Ramosyza E (2011). The effect of osteoarthritis definition on prevalence and incidence estimates: a systematic review. *Osteoarthritis and Cartilage*.

[2] Carr AJ, Robertsson O, Graves S (2012). Knee replacement. *The Lancet*.

[3] Affas F, Nygårds EB, Stiller CO, Wretenberg P, Olofsson C (2011). Pain control after total knee arthroplasty: a randomized trial comparing local infiltration anesthesia and continuous femoral block. *Acta Orthopaedica*.

[4] Brown AK, Christo PJ, Wu CL (2004). Strategies for postoperative pain management. *Best Practice and Research*.

[5] Chung JWY, Lui JCZ (2003). Postoperative pain management: study of patients’ level of pain and satisfaction with health care providers’ responsiveness to their reports of pain. *Nursing and Health Sciences*.

[6] Yates P, Dewar A, Edwards H, Fentiman B, Najman J, Nash R (1998). The prevalence and perception of pain amongst hospital in-patients. *Journal of Clinical Nursing*.

[7] Watt-Watson J, Stevens B, Garfinkel P, Streiner D, Gallop R (2001). Relationship between nurses’ pain knowledge and pain management outcomes for their postoperative cardiac patients. *Journal of Advanced Nursing*.

[8] Rosenquist RW, Rosenberg J (2003). Postoperative pain guidelines. *Regional Anesthesia and Pain Medicine*.

[9] Beers MH (2006). *The Merck Manual of Diagnostics and Therapy*.

[10] Ahles TA, Tope DM, Pinkson B (1999). Massage therapy for patients undergoing autologous bone marrow transplantation. *Journal of Pain and Symptom Management*.

[11] NIH Consensus Development Panel of Acupuncture (1998). NIH consensus conference: acupuncture. *Journal of the American Medical Association*.

[12] Sun Y, Gan TJ, Dubose JW, Habib AS (2008). Acupuncture and related techniques for postoperative pain: a systematic review of randomized controlled trials. *British Journal of Anaesthesia*.

[13] Manheimer E, Earon K, Lao L, White AR, Morozova O (2009). *Acupuncture for the Treatment of Post-Operative Pain (Protocol)*.

[14] Lin JG, Chen WL (2008). Acupuncture analgesia: a review of its mechanisms of actions. *American Journal of Chinese Medicine*.

[15] Usichenko TI, Dinse M, Hermsen M, Witstruck T, Pavlovic D, Lehmann C (2005). Auricular acupuncture for pain relief after total hip arthroplasty—a randomized controlled study. *Pain*.

[16] Usichenko TI, Hermsen M, Witstruck T (2005). Auricular acupuncture for pain relief after ambulatory knee arthroscopy—a pilot study. *Evidence-Based Complementary and Alternative Medicine*.

[17] Usichenko TI, Kuchling S, Witstruck T (2007). Auricular acupuncture for pain relief after ambulatory knee surgery: a randomized trial. *Canadian Medical Association Journal*.

[18] Usichenko TI, Lehmann C, Ernst E (2008). Auricular acupuncture for postoperative pain control: a systematic review of randomised clinical trials. *Anaesthesia*.

[19] Huang LC (2005). *Auricular Treatment—Formulate and Prescriptions*.

[20] Auricular points—subcortex, antitragic aex, middle border, brain stem.

[21] Yan LP, Li H (2001). Analysis of clinical application of the ear Shenmen. *Shanghai Journal of Traditional Chinese Medicine*.

[22] Katz J, Melzack R (1999). Measurement of pain. *Surgical Clinics of North America*.

[23] Strassels SA, McNicol E, Suleman R (2005). Postoperative pain management: a practical review—part 1. *American Journal of Health-System Pharmacy*.

[24] Melzack R (1987). The short-form McGill pain questionnaire. *Pain*.

[25] Chang MY, Chen CH, Huang KF (2006). A comparison of massage effects on labor pain using the McGill Pain Questionnaire. *The Journal of Nursing Research*.

[26] Kerr DR, Kohan L (2008). Local infiltration analgesia: a technique for the control of acute postoperative pain following knee and hip surgery—a case study of 325 patients. *Acta Orthopaedica*.

[27] Norkin CC, White J, Chang YHH (1996). *Measurement of Joint Motion—A Guide to Goniometry*.

[28] Han J-S (2011). Acupuncture analgesia: areas of consensus and controversy. *Pain*.

[29] Tsang RCC, Tsang PL, Ko CY, Kong BCH, Lee WY, Yip HT (2007). Effects of acupuncture and sham acupuncture in addition to physiotherapy in patients undergoing bilateral total knee arthroplasty—a randomized controlled trial. *Clinical Rehabilitation*.

